# Chicken volatiles repel host-seeking malaria mosquitoes

**DOI:** 10.1186/s12936-016-1386-3

**Published:** 2016-07-21

**Authors:** Kassahun T. Jaleta, Sharon Rose Hill, Göran Birgersson, Habte Tekie, Rickard Ignell

**Affiliations:** Unit of Chemical Ecology, Department of Plant Protection Biology, Swedish University of Agricultural Sciences, P. O. Box 102, 23053 Alnarp, Sweden; Department of Zoological Sciences, College of Natural Science, Addis Ababa University, P. O. Box 1176, Addis Ababa, Ethiopia

**Keywords:** Host discrimination, Host species abundance, Blood meal analysis, Non-host volatiles, *Anopheles arabiensis*

## Abstract

**Background:**

*Anopheles arabiensis* is a dominant vector of malaria in sub-Saharan Africa, which feeds indoors and outdoors on human and other vertebrate hosts, making it a difficult species to control with existing control methods. Novel methods that reduce human-vector interactions are, therefore, required to improve the impact of vector control programmes. Investigating the mechanisms underlying the host discrimination process in *An. arabiensis* could provide valuable knowledge leading to the development of novel control technologies. In this study, a host census and blood meal analysis were conducted to determine the host selection behaviour of *An. arabiensis*. Since mosquitoes select and discriminate among hosts primarily using olfaction, the volatile headspace of the preferred non-human host and non-host species, were collected. Using combined gas chromatography and electroantennographic detection analysis followed by combined gas chromatography and mass spectrometry, the bioactive compounds in the headspace collections were identified. The efficiency of the identified non-host compounds to repel host-seeking malaria mosquitoes was tested under field conditions.

**Results:**

The host census and blood meal analyses demonstrated that *An. arabiensis* strongly prefers human blood when host seeking indoors, while it randomly feeds on cattle, goats and sheep when found outdoors. However, *An. arabiensis* avoids chickens despite their relatively high abundance, indicating that chickens are a non-host species for this vector. Eleven bioactive compounds were found in the headspace of the non-host species. Six of these were species-specific, out of which four were identified using combined gas chromatography and mass spectrometry. When tested in the field, the chicken-specific compounds, isobutyl butyrate, naphthalene, hexadecane and *trans*-limonene oxide, and the generic host compounds, limonene, *cis*-limonene oxide and *β*-myrcene, significantly reduced trap catches within the house compared to a negative control. A significant reduction in trap catch was also observed when suspending a caged chicken next to the trap.

**Conclusions:**

Non-host volatiles repel host-seeking *An. arabiensis* and thus play a significant role in host discrimination. As such, this study demonstrates that non-host volatiles can provide protection to humans at risk of mosquito-vectored diseases in combination with established control programmes.

**Electronic supplementary material:**

The online version of this article (doi:10.1186/s12936-016-1386-3) contains supplementary material, which is available to authorized users.

## Background

Despite recent global intervention efforts, malaria remains a major public health problem in sub-Saharan Africa [1–4]. The widespread use of indoor residual spraying (IRS) and insecticide-treated bed nets (ITNs) has led to a significant reduction in the main vector of malaria, *Anopheles gambiae* sensu stricto, throughout much of sub-Saharan Africa [5, 6]. However, the integrated IRS/ITN strategy has inadvertently led to a proportional shift to outdoor residual malaria transmission by sympatric species, in particular *Anopheles arabiensis*, which is now a dominant malaria vector in the region [7–10]. As *An. arabiensis* is an opportunistic feeder on both human and other vertebrate hosts [11–14], its ability to feed indoors and outdoors on available hosts, makes this mosquito a vector that requires a more coordinated control strategy [7, 13, 14]. After the introduction and continued use of IRS and ITNs, *Anopheles* mosquito populations have been reported to change from feeding indoors to feeding outdoors [6, 9, 15]. This has resulted in a change in the proportion of females that feed on human blood [10], and thus has altered the malaria transmission dynamics [16, 17]. The behavioural plasticity in host choice, demonstrated by either an individual or a population, is likely constrained by the mosquitoes’ host preference that delineates a hierarchy of acceptable blood hosts [14, 18]. Understanding the mechanisms underlying the host discrimination process in *An. arabiensis* may guide the development of new vector control strategies based on sustained modification of mosquito behaviour.

Host selection in mosquitoes is determined by both intrinsic and extrinsic factors [14, 18]. One important extrinsic factor is the availability of host species, which may be a crucial determinant of host choice, especially for opportunistic mosquito species [14, 18, 19]. The forage ratio assesses the dependence of host choice on host availability by comparing the proportion of blood meals from a particular host species with their relative abundance in the environment [20]. For example, the proportion of *An. arabiensis* female mosquitoes that blood feed on humans is higher in indoor-caught mosquitoes, and in the absence of cattle in the surrounding area [21, 22]. Host choice in *An. arabiensis*, however, does not always overlap with host availability, as the species appears to have a low preference for birds, regardless of their abundance [22–25]. This discrimination suggests that *An. arabiensis* has evolved mechanisms to differentiate between potential host species.

*Anopheles* mosquitoes primarily use their sense of smell to locate suitable hosts. Qualitative differences in the detected volatile profiles associated with the various hosts provide a chemical signature on which female host selection relies [26]. Different combinations of these volatile host-related attractants have been employed in the development of bait technologies for the control of *Anopheles* mosquitoes [27]. Research on herbivorous and other blood-feeding insects also indicates that host choice involves repellents, so-called non-host volatiles (NHVs) that act together with host attractants during host discrimination [28–31]. NHVs can be exploited for the manipulation of blood-feeding insects, as shown for example in the Morsitans group of tsetse flies, *Glossina* spp., which transmit trypanosomiasis (nagana) in cattle [29–31].

Through vertebrate host abundance and blood meal analyses, multiple hosts and a single non-host species of field-caught *An. arabiensis* were identified. A comparison of the olfactory responses of female *An. arabiensis* to volatile headspace extracts collected from the non-human hosts and the non-host revealed both generic and species-specific compounds. Based on the combined results of these analyses, this study hypothesized that specific compounds identified in the volatile extract of the non-host constitute a protective chemical barrier. This hypothesis was tested by evaluating the response of host-seeking *An. arabiensis*, to identify NHVs in field trials.

## Methods

### Population data on potential host species

Data on the population of human and domestic animals from three villages, Wama Kusaye (8°58.695′N, 36°48.558′E; 1443 m above sea level), Baka-Boro (8°57.715′N, 36°52.058′E; 1522 m above sea level) and Machara (8°58.028′N, 36°42.994′E; 1514 m), in the East Wollega Zone of western Ethiopia was obtained from agricultural extension workers and the local administration office. The common practice in this region is for livestock and people to share their living quarters, and as such, the assumption was made that the availability of potential hosts is similar both indoors and outdoors.

### Mosquito collection and blood meal analysis

Blood-fed mosquitoes were collected from the three villages on five separate days, using standard collection methods [32]. Indoor resting mosquitoes were collected in ten houses, in each village, from 06:00 to 08:00. Mosquito-knockdown collections were performed by spraying with Kilit™ (Miswa Chemicals Ltd, UK), a synthetic pyrethrum. Outdoor-resting mosquitoes were surveyed at five pit shelters dug for the purpose (1.5 × 1.0 × 2.0 m, with horizontal ‘pockets’ dug in the four walls of each) [32] in each village.

*Anopheles* mosquitoes were counted and then sorted by sex, abdominal condition (unfed, freshly fed, half gravid and gravid), and species using morphological keys [33]. The *Anopheles* mosquitoes that were provisionally identified as *An. gambiae* s.l., were screened using polymerase chain reaction (PCR) described by Scott et al. [34] and conclusively identified.

Freshly blood-fed mosquitoes were cut transversely between the thorax and the abdomen, and the posterior portions containing the blood meal were tested for source host blood by the direct enzyme-linked immunosorbent assay (ELISA) [35]. Commercially available anti-host (IgG) conjugates against human, cattle, goat, sheep and chicken (Kirkegard and Perry Laboratories, MD, USA) were used in the ELISA. Control samples consisted of blood drawn from a human (KTJ), and blood obtained from cow, sheep and goat (Addis Ababa Abattoirs enterprise), as well as chicken blood obtained from a local restaurant. Each mosquito was tested simultaneously for human, cattle, goat, sheep, and chicken antibodies. Significant differences in blood meals found in indoor- and outdoor-resting mosquitoes were determined using Chi squared (***χ***^2^) analyses (Prism v. 5, GraphPad, CA, USA).

### Forage ratio

The forage ratio was calculated as the proportion of host species present in blood meals of *An. arabiensis* divided by the proportion of host species available in the environment [36].

### Volatile headspace collections

Headspace collections were obtained from cows, sheep, goats, and chicken. For this purpose, at least five individuals of each species were randomly selected from the Wama Kusaye village. The host hair, wool or feathers were cut with sterilized scissors, enclosed in separate polyacetete bags (Toppits, Melitta, Sweden) and immediately transported to the laboratory. The mixed hair, wool or feathers (20 ± 1 g) were placed in a glass wash bottle. A charcoal-filtered, continuous airstream (100 ml min^−1^) was drawn by a diaphragm vacuum pump (KNF Neuberger, Freiburg, Germany) through the bottle onto an aeration column for 24 h. The aeration column consisted of a Teflon tube (4 mm diameter × 40 mm length) holding 30 mg Porapak Q (80/100 mesh, Alltech, Deerfield, IL, USA) between polypropylene wool plugs. Adsorbed volatiles were desorbed by eluting each column with 500 µl of re-distilled *n*-hexane (≥99.9 % purity, Merck KGaA, Darmstadt, Germany) and condensed under N_2_ to approximately one-quarter of the volume. Samples were stored at −20 °C.

### Mosquito rearing

*Anopheles arabiensis* (Dongola strain) were maintained at 27 ± 2 °C, 70 ± 2 % relative humidity and at a light:dark cycle of 12:12 h. Larvae were reared in plastic trays (20 × 18 × 7 cm) and fed Tetramin™ fish food (Tetra, Melle, Germany). Pupae were transferred to Bugdorm cages (30 × 30 × 30 cm, MegaView Science, Taiwan) for adults to emerge. Adults were provided 10 % sucrose solution ad libitum. For colony maintenance, female mosquitoes were provided with sheep blood (Håtunalab, Bro, Sweden) using an artificial feeder (Hemotek, Discovery Workshops, Accrington, UK). Electrophysiological analysis was conducted on four- to six-day post-emergence non-blood fed female mosquitoes.

### Electrophysiology

Antennal responses to the headspace volatile collections were examined by combined gas chromatography (GC) and electroantennographic detection (EAD) analysis as well as electro-antennography (EAG) using an EAG system (IDAC-2; Syntech, Kirchgarten, Germany) and an Agilent 6890 N GC (Agilent Technologies, Santa Clara, CA, USA). For the GC-EAD analysis, the GC was equipped with a HP-5MS (Agilent Technologies) fused silica capillary column (30 m × 0.25 mm; df = 0.25 µm). Hydrogen was used as mobile phase (Q = 45 cm s^−1^). Two µl of each sample were injected (splitless mode, 30 s, injector temperature 225 °C). The GC oven temperature gradient was programmed from 30 °C (4-min hold) at 8 °C min^−1^ to 250 °C (5-min hold). To the GC effluent, 4 psi of nitrogen was added and split 1:1 in a Gerstel 3D/2 low dead volume four way-cross (Gerstel, Mülheim, Germany) between the flame ionization detector and the EAD. The GC effluent capillary for the EAD passed through a Gerstel olfactory detection port-2 transfer line, which mirrored the GC oven temperature, into a glass tube (8 mm diameter × 10 cm length), where it was mixed with charcoal-filtered, humidified air (1 l min^−1^). The antenna was placed 0.5 cm from the outlet of this tube.

For EAG recordings, the excised head of a female *An. arabiensis* was used. After removing the distal tip of the first flagellomere of one antenna, it was inserted into a recording glass electrode filled with Beadle-Ephrussi ringer (140 mM NaCl, 4.7 mM KCl, 1.9 mM CaCl_2_·2H_2_O) and connected to a pre-amplifier (10×) probe connected to a high impedance DC amplifier interface box (IDAC-2; Syntech). The indifferent electrode was inserted into the occipital foramen. At least six GC-EAD runs were made for each headspace volatile collection on different preparations.

### Chemical analysis

Volatile collections were analysed on a combined gas chromatography and mass spectrometer (GC–MS) (6890 GC and 5975 MS; Agilent Technologies) operated in the electron impact ionization mode at 70 eV. The GC was equipped with a similar column as for the GC-EAD analysis. Helium was used as the mobile phase (Q = 35 cm s^−1^). The GC oven temperature was programmed as for the GC-EAD analysis above. Compounds were identified according to their Kovat’s indices and mass spectra in comparison with custom made and NIST-05 libraries, and confirmed by co-injection of authentic standards (Additional file 1).

### Dose–response experiments

For further verification of the physiological activity of the chemicals identified through GC-EAD and GC-MS analyses, dose–response experiments were conducted by EAG recordings using synthetic standards (Additional file 1). Concentrations ranged in decadic steps from 0.001 to 10 % (volume/volume) for each synthetic compound. Dilutions of compounds were prepared in redistilled *n*-hexane (LabScan, Malmö, Sweden), except for furfuryl alcohol for which absolute ethanol was used (LabScan). Odour stimuli were produced by loading 10 µl of each diluted synthetic test compound onto a filter paper (1 × 1.5 cm, Munktell Filter AB, Sweden) inserted inside a glass Pasteur pipette. Pipettes with formulated filter papers were kept for 30 min in a fume hood prior to use to allow for solvent evaporation. The pipette was connected via a silicone tube to a stimulus generator (CS-55; Syntech) and the tip of the pipette was inserted into the glass tube with an air flow (1 l min^−1^) directed towards the antenna. Stimuli were produced by puffing air (0.5 l min^−1^) through the pipette during 0.5 s; each pipette was used only once. Hexane was used as a solvent blank, as the first and last stimulus for every replicate, except ethanol that was used as a solvent blank for furfuryl alcohol. Each set of odour stimuli was tested on one antenna (n = 6). The responses to each test stimulus were calculated by subtracting the averaged response amplitude of the solvent controls from the response amplitude of the stimulus.

### Field evaluation of identified host and non-host volatiles

Field experiments were conducted in the Wama Kusaye village. In the village, 11 thatched houses were selected based on similarities in size, with houses separated approximately 200 m apart. The experimental design followed a Latin square, in which treatments were randomly assigned to houses on the first day and then rotated between houses to minimize location bias over the following days, for a total of 11 days. The experiments were conducted in November and December 2012, i.e., after the long rainy season, when host-seeking *An. arabiensis* were readily available. In each house, a single volunteer (27–36 years old) slept under an untreated bed net. A Centers for Disease Control and Prevention (CDC) mini-light trap (BioQuip Products, Inc, CA, USA), with the light bulb removed, was hung next to the foot of the bed net, approximately 1 m above ground level. Ethical clearance was obtained from the Ethical Committee of the Faculty of Science, Addis Ababa University conforming to the WMA Declaration of Helsinki.

**Fig. 1:**
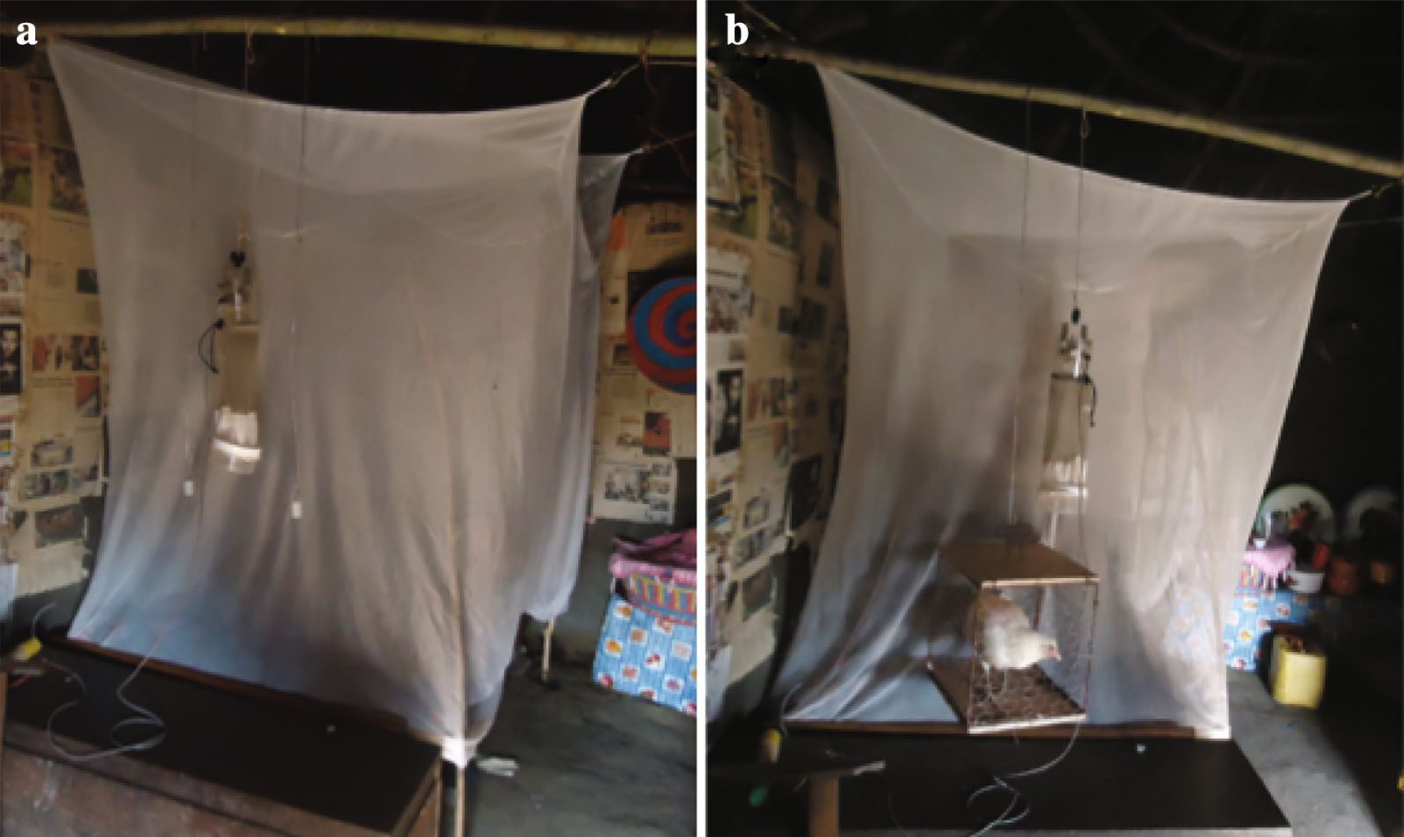
CDC suction traps used in the field experiment were placed at the foot of a bed with a volunteer sleeping under a bed net. Dispenser vials, releasing test compounds at a rate of 1 mg h^−1^, were suspended next to the traps (**a**). As a control, a live caged chicken was used in lieu of the dispenser (**b**)

Synthetic compounds of nine of the GC-EAD active compounds identified in the volatile headspace collections of the non-host (chicken) and hosts (cattle, goats, and sheep) of *An. arabiensis* were used in the study. Dispenser vials (PE# 733, Kartell, Italy), each containing 0.5 g of a synthetic compound released at a rate of 1 mg h^−1^, were suspended approximately 10 cm beside and 20 cm below the trap using wire hooks (Fig. 1). The required release rate was achieved by varying the number of caps attached to each trap, and the size of the hole in the cap from which the chemical could volatilize. The number of caps and hole size required was determined: full caps were weighed and reweighed after 1, 2, 3, 4, 5, 6, 12, and 24 h of exposure to field conditions (25 ± 1 °C, 60 % RH). This procedure was repeated six times to calculate an average release rate for each compound. As a negative control, a similar trap, with solvent alone, was used. In addition, a caged chicken surrounded by a fine mesh screen, to prevent chicken-mosquito interactions, and suspended in a similar way as the dispensers, served as a control (Fig. 1). The traps were turned on at 18:00 and turned off the following morning at 06:00. Caught mosquitoes were enumerated and identified to species, as described above. The effect of compounds on the number of mosquitoes caught (distributed response variable) was subjected to a generalized linear mixed effect model procedure (GLMM, lmer) in the R statistical software version 3.1.1. (“house” and “day” were controlled for as random effects). The model used a Poisson distribution and log-link function for its construction, and AIC was used for model evaluation. For a comparative analysis among the different compounds, a posthoc test, adjusted for multiple comparisons, was performed on a linear mixed effects model (R, lme4, multcomp; Chi squared, ***χ***^2^; *P* < 0.05).

## Results

### Mosquito species identification and composition

Four species of *Anopheles* mosquitoes, *An. arabiensis,Anopheles funestus* s. l.*, Anopheles nili* and *Anopheles coustani*, were collected and identified in the study villages (n = 4844). *Anopheles arabiensis*, as determined by PCR analysis of 386 mosquitoes (more than 5 % of the mosquitoes caught), was the most abundant species, comprising more than 98.5 % of the total mosquitoes caught. A total of 4739 female *An. arabiensis* were collected from the study villages, using pyrethrum spray sheet collections (n = 1036, 758 and 503 for Wama Kussaye, Baka-Boro and Machara, respectively) and artificial pit shelters (n = 1264, 639 and 539 for Wama Kussaye, Baka-Boro and Machara, respectively). During the field evaluation of the non-host volatiles, two species of mosquitoes, *An. arabiensis* and *An. coustani*, were collected and identified. *Anopheles arabiensis*, as determined by PCR, was the most abundant species comprising more than 97 % of the total mosquitoes caught (n = 583).

### Host-species abundance and feeding preference of *Anopheles arabiensis*

Preferred and non-preferred host species of *An. arabiensis* were identified through analyses of host abundance and blood meal prevalence (Table 1). Cattle were the most abundant host species in all villages, with humans one-third and chickens two-thirds less abundant. The number of females feeding on human and cattle hosts differed significantly between indoor and outdoor events (***χ***^**2**^ = 186.7, *P* < 0.0001; ***χ***^**2**^ = 18.8, *P* < 0.001, respectively; Table 1). Calculated forage ratios (Table 1) showed a high preference of *An. arabiensis* for humans as a source of a blood meal when collected indoors (>2), but a low preference, with a forage ratio <1 indicative of avoidance, when collected outdoors. An almost diametrically opposite forage ratio was found for cattle indoors, indicating that *An. arabiensis* actively avoid cattle when searching for a blood meal indoors. For goat and sheep, the calculated forage ratio was ca. 1 indicating that *An. arabiensis* randomly feed on these hosts both indoors and outdoors. Interestingly, the calculated forage ratio for chicken (0) indicates that chickens are a non-host, despite its relatively high abundance.

**Table 1:** Host availability, blood meal analyses, and forage ratio of *Anopheles arabiensis*

Hosts	Host availability	Blood meal				Forage ratio	
		Indoor		Outdoor		Indoor	Outdoor
	No.	No.	%	No.	%		
Human	6706	523	69	81	20	2.2	0.6
Cattle	9970	139	18	260	63	0.4	1.3
Goat	849	25	3.3	21	5	0.8	1.3
Sheep	481	15	2	11	2.6	0.9	1.1
Chicken	3194	0	0	1	0.2	0	0
Mixed	–	39	5.2	26	6.3	–	–
Unidentified	–	15	2	16	3.8	–	–

Host availability is denoted by number of host individuals (No.) present in the three villages. Blood meals were analysed and reported as both number of individual events (No.) and percent (%) of the total number of individuals feeding on a particular host

### GC-EAD and GC-MS analyses of headspace volatile collections

A total of 25 GC-EAD active compounds were identified in the headspace volatile collections from the non-human hosts: cattle, sheep and goat (Table 2). Four of these compounds co-occurred in all of the collections, while nine compounds co-occurred in two of the three collections. The generic compounds identified in the headspace volatile collections of all non-human host species included limonene, nonanal, phenyl acetaldehyde, and sulcatone. Species-specific compounds included 2-butoxyethanol, *E*2-heptenal, neral, and furfuryl alcohol in cattle; benzyl alcohol and heptanal in goat; and 1-methylnaphthalene, *p*-cymene, *m*-propylphenol, and *cis*-dihydrocarvone in sheep.

**Table 2:** Physiologically active compounds identified through GC-EAD and GC-MS analyses of odours collected from hair, wool and feathers of hosts (cow, goat and sheep) and non-host (chicken) of *Anopheles arabiensis*

Compounds	Cow	Goat	Sheep	Chicken
Hydrocarbons				
Aliphatics				
Hexadecane	–	–	–	x
Aromatics				
Naphthalene	–	–	–	x
1-Methylnaphthalene	–	–	x	–
Monoterpenes				
Limonene	x	x	x	x
*β*-Myrcene	x	–	–	x
*p*-Cymene	–	–	x	–
Alcohols				
Aliphatics				
2-Butoxyethanol	x	–	–	–
Octanol	x	–	x	–
Aromatics				
Benzyl alcohol	–	x	–	–
*o*-Cresol	x	–	x	–
*m*-Cresol	x	x	–	–
*p*-Cresol	x	–	x	–
*m*-propylphenol		–	x	–
Monoterpenes				
Linalool	x	–	x	–
Aldehydes				
Aliphatics				
Heptanal	–	x	–	–
*E*2-Heptenal	x	–	–	–
*E*2-Octenal	x	x	–	–
Nonanal	x	x	x	x
*E*2-Nonenal	x	x	–	–
Aromatics				
Benzaldehyde	–	x	x	–
Phenyl acetaldehyde	x	x	x	–
Monoterpenes				
Neral	x	–	–	–
Ketones				
Monoterpenes				
*cis*-Dihydrocarvone	–	–	x	–
Irregular terpenes				
Sulcatone	x	x	x	x
Esters				
Aliphatics				
Isobutyl butanote	–	–	–	x
Others				
Monoterpenes				
*cis*-Limonene oxide	–	x	x	x
*trans*-Limonene oxide	–	–	–	x
Heterocyclics				
Furfuryl alcohol	x	–	–	–
Unknowns				
Unknown 1	–	–	–	x
Unknown 2	–	–	–	x

Detection (x) and lack of detection (–) of a compound by GC-EAD are indicated

In the headspace volatile collection from the non-host, chicken, 11 GC-EAD active compounds were detected (Table 2). Of these, limonene, *β*-myrcene, nonanal, sulcatone and *cis*-limonene oxide were also found within volatile collections of one or more of the non-human hosts. The remaining compounds, hexadecane, naphthalene, isobutyl butanoate and *trans*-limonene oxide, were specific to chicken. This study was unable to confirm the identity of two chicken-specific compounds using commercially available synthetic standards and are here referred to as unknown 1 and 2.

For further verification of the physiological activity of the compounds identified through GC-EAD and GC-MS analyses, dose–response experiments were conducted by EAG recordings using synthetic standards (Additional file 1). The EAG dose–response analysis of the GC-EAD active compounds demonstrated that *An. arabiensis* respond to all tested synthetics in a dose-dependent manner, and confirmed that the antennae were differentially sensitive to these compounds (Additional file 2).

### Field evaluation of non-host and generic volatiles

**Fig. 2:**
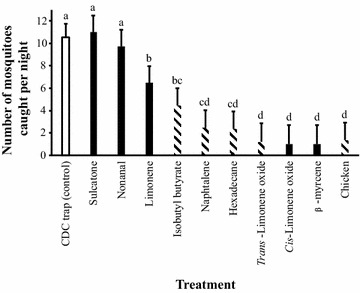
The mean number (±SEM) of host-seeking *Anopheles arabiensis* caught in CDC suction traps baited with synthetic chicken-specific (*hatched bars*) and generic (*solid bars*) host compounds or a live chicken (*hatched bar*) compared to a CDC control trap (*open bar*). The mean mosquito catches per treatment sharing the same letter designation are not significantly different from one another (generalized linear model; *P* > 0.05)

Overall, the tested volatiles had a significant effect on trap catches when tested in the field using suction CDC traps (CDC traps without light; $\varvec{\chi}_{10}^{2}$ = 226.76, *P* < 0.001; Figs. 1 and 2). Traps baited individually with the chicken-specific volatiles, isobutyl butanoate, naphthalene, hexadecane and *trans*-limonene oxide, and with the generic compounds, limonene, *cis*-limonene oxide and *β*-myrcene, caught significantly fewer *An. arabiensis* compared to the solvent baited negative control trap (Fig. 2). Similarly, a significantly lower number of mosquitoes were caught in a trap baited with a live, caged chicken (Fig. 2). In contrast, CDC traps baited with either of the generic compounds, sulcatone or nonanal, did not affect the number of *An. arabiensis* caught, compared to the solvent baited negative control trap (Fig. 2).

## Discussion

*Anopheles arabiensis* is a selective blood feeder when host-seeking indoors, which prefers human blood and avoids cattle blood. In contrast, when found outdoors, *An. arabiensis* is an opportunistic blood feeder, randomly feeding on cattle, goats and sheep and avoiding humans. The breadth of the host range suggests that the use of alternative hosts may be important in maintaining the local mosquito population density, and thus affects the risk of malaria transmission. While *An. arabiensis* feeds on many abundant vertebrate species, this study shows that it avoids chickens despite their relatively high abundance. These results are consistent with previous studies [22–25], implying that *An. arabiensis*, although opportunistic, exhibits non-random feeding on available hosts. The selective advantage of such behaviour may be explained by variation in nutritional rewards and the corresponding fitness accruing from feeding on different host types [18]. Variation in physical and chemical properties of the blood between host species may be a driver for the evolution of host-choice in *An. arabiensis.* This could be a factor contributing to the avoidance of chicken as a host [18, 37]. Additional factors that can influence the feeding success of mosquitoes are the physical barrier to mosquito feeding provided by the feathers as well as the chicken’s prey behaviour, since the birds will actively feed on mosquitoes.

Volatile compounds identified in the headspace extracts of chicken feathers appear to play a pivotal role in the observed non-host avoidance. Compounds that were able to disrupt the host-seeking behaviour of *An. arabiensis* included both chicken-specific and generic volatiles. This suggests that these volatiles function as medium- to long-range repellents. Of the identified compounds, naphthalene has previously been found at higher levels in ‘non-attractive’ individuals of humans and cattle, in which it appears to either repel or mask the response of the biting midge *Culicoides impunctatus* [38] and cattle flies [39] to normally attractive compounds. Limonene oxides and *β*-myrcene have not been identified previously in the odour profile of vertebrates, but are known to be botanical insect repellents [40, 41]. Although many studies have demonstrated that haematophagous insects show feeding preferences for certain host species, this is the first to implicate non-host volatiles in the differential host attractiveness to mosquitoes. Previous work on tsetse flies, however, suggests that NHVs are an important part of the host selection process in haematophagous insects [31]. The adaptive value of the behavioural response to NHVs is likely linked to higher fecundity and survival after feeding on preferred host species than on non-preferred hosts [18].

Non-host volatiles, acting either as repellents or masking agents, can be developed to be used in concert with established integrated vector management programs. Proof of principle for this has been shown for tsetse flies, where compounds identified in waterbuck act as potent non-host repellents [31]. Moreover, compounds identified in a non-host fish, turbot, when added to salmon-conditioned water, have been shown to interfere with the host-seeking behaviour of the salmon louse [42].

## Conclusions

This study demonstrates that NHVs have the potential to afford protection to people at risk of contracting a mosquito-vectored disease, in combination with established control programmes. Future work will be aimed at determining the efficacy and duration of protection of a spatial repellent product formulated according to WHO’s guidelines [43]. With the increasing reports on insecticide resistance among disease vectors, it is incumbent on the international malaria community to embrace these novel control methods and products.

## Additional files

10.1186/s12936-016-1386-3 Synthetic compounds used for the verification of physiologically active compounds in the natural headspace extracts of cattle hair, sheep wool, goat hair and chicken feathers.
10.1186/s12936-016-1386-3 Antennal responses of female *Anopheles arabiensis* to various doses of compounds identified in the headspace of chicken feathers, cattle hair, goat hair and sheep wool. Error bars represent the standard error of the mean (n = 6).

## Abbreviations

CDC: Centers for Disease Control and Prevention
EAD: electroantennographic detection
EAG: electroantennography
ELISA: enzyme-linked immunosorbent assay
GC: gas chromatograph
IRS: indoor residual spraying
ITN: insecticide-treated bed net
MS: mass spectrometry
NHV: non-host volatile
GLMM: generalized linear mixed effect model
