# Supplementary material for: Chicken volatiles repel host-seeking malaria mosquitoes
Source: Malar J. 2016 Jul 21;15:354. doi: 10.1186/s12936-016-1386-3 (PMC4955153; doi:10.1186/s12936-016-1386-3)
Supplement: Supplementary file 1 — 10.1186/s12936-016-1386-3 Synthetic compounds used for the verification of physiologically active compounds in the natural headspace extracts of cattle hair, sheep wool, goat hair and chicken feathers. [file 12936_2016_1386_MOESM1_ESM.pdf]

Compounds	Systematic name	Purity (%)	CAS number	Company
**Hydrocarbons**				
**Aliphatics**				
Hexadecane	Hexadecane	99	544-76-3	Sigma-Aldrich
**Aromatics**				
Naphthalene	Bicyclo[4.4.0]deca-1,3,5,7,9-pentene	99	91-20-3	Sigma-Aldrich
1-Methylnaphthalene	1-Methyl-bicyclo[4.4.0]deca-1,3,5,7,9-pentene	97	90-12-0	Sigma-Aldrich
**Monoterpenes**				
Limonene	1-Methyl-4-(1-methylethyl)-1-cyclohexene	92	5989-54-8	Acros Organics
β-Myrcene	7-methyl-3-methylene-1,6-octadiene	90	123-35-3	INC Biomedicals Inc.
p-Cymene	1-Methyl-4-(1-methylethyl)-benzene	99	99-87-6	ACROS
**Alcohols**				
**Aliphatics**				
2-Butoxyethanol	2-Butoxy-ethanol	98	111-76-2	Fluka
Octanol	1-Octanol	99	111-87-5	Sigma-Aldrich
**Aromatics**				
Benzyl alcohol	Phenyl methanol	99	100-51-6	Sigma-Aldrich
o-Cresol	2-Methyl phenol	99	95-48-7	Aldrich
m-Cresol	3-Methyl phenol	97	108-39-4	Aldrich
p-Cresol	4-Methyl phenol	99	104-44-5	Aldrich
m-propylphenol	3-Propyl phenol	98	621-27-2	Fluka
**Monoterpenes**				
Linalool	3,7-Dimethylocta-1,6-dien-3-ol	97	78-70-6	Aldrich
**Aldehydes**				
**Aliphatics**				
Heptanal	Heptanal	95	111-71-7	Aldrich
*E*2-Heptenal	(2*E*)-2-Heptenal	95	18829-55-5	SAFC
*E*2-Octenal	(2*E*)-2-Octenal	94	2548-87-0	SAFC
Nonanal	Nonanal	95	124-19-6	Acros Organics
*E*2-Nonenal	(2*E*)-2-Nonenal	99	18829-56-6	Sigma-Aldrich
**Aromatics**				
Benzaldehyde	Phenyl methanal	99.5	100-52-7	Fluka
Phenyl acetaldehyde	2-Phenyl ethanal	95	122-78-1	Sigma-Aldrich
**Monoterpenes**				
Neral	(2*E*)-3,7-Dimethyl-2,6-octadienal	95	106-26-3	Sigma-Aldrich
**Ketones**				
**Monoterpenes**				
cis-Dihydrocarvone	*cis*-Menth-8(10)-en-2-one	97	5524-05-0	Aldrich
**Irregular Terpenes**				
Sulcatone	6-Methyl-5-hepten-2-one	99	110-93-0	Aldrich
**Esters**				
**Aliphatics**				
Isobutyl butanoate	2-Methylpropyl butanoate	98	539-90-2	SAFC
**Others**				
**Monoterpenes**				
cis-Limonene oxide	*cis*-Limonene 1,2-epoxide [#)]	97	1195-92-2	Sigma-Aldrich
trans-Limonene oxide	*trans*-Limonene 1,2-epoxide [#)]	95	6909-30-4	Sigma-Aldrich
**Heterocyclics**				
Furfuryl alcohol	2-furan methanol	98	98-00-0	Aldrich

[#)] Limonene = 1-Methyl-4-(1-methylethyl)-1-cyclohexene
