# Supplementary figures and images for: Chicken volatiles repel host-seeking malaria mosquitoes
Source: Malar J. 2016 Jul 21;15:354. doi: 10.1186/s12936-016-1386-3 (PMC4955153; doi:10.1186/s12936-016-1386-3)

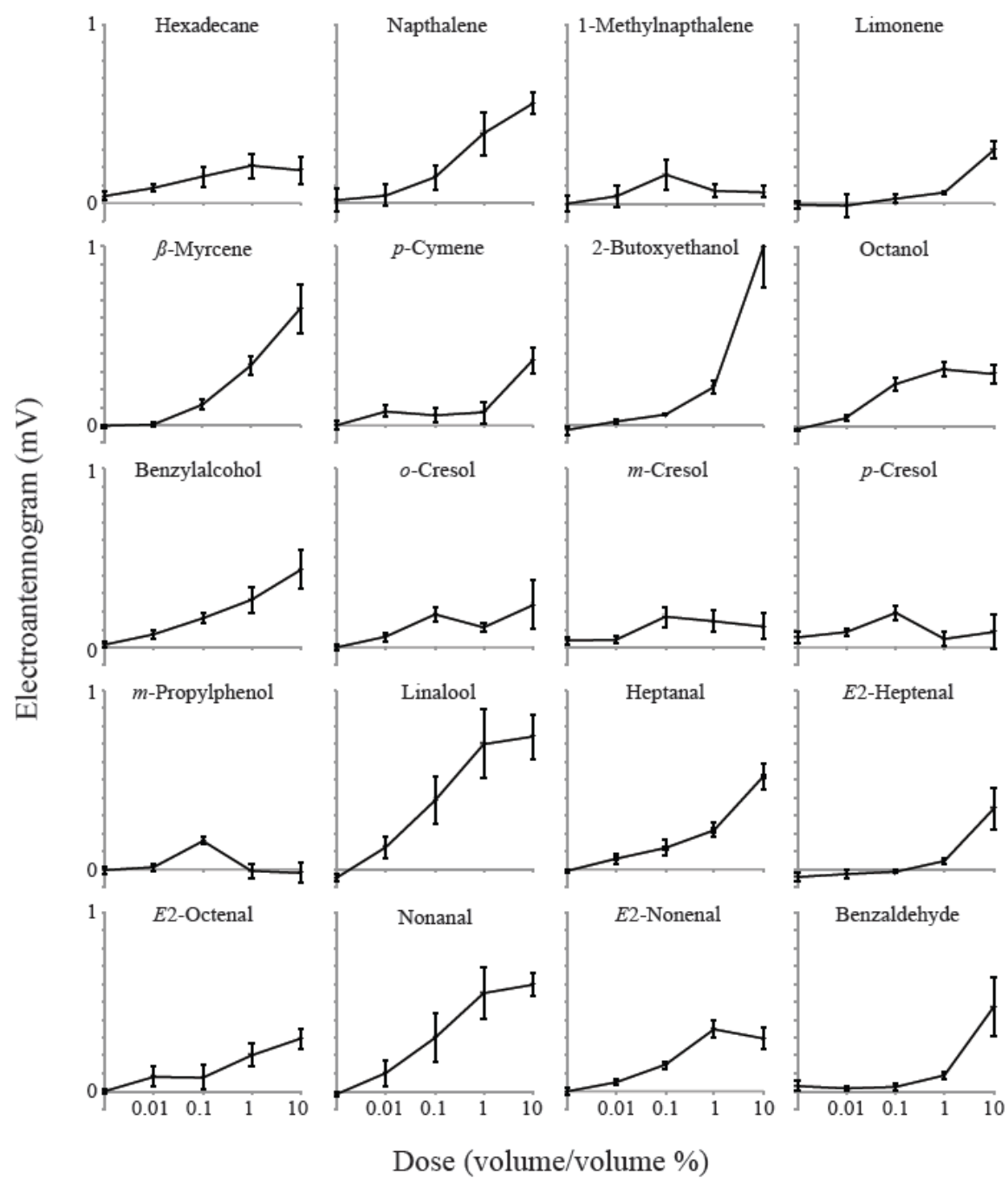

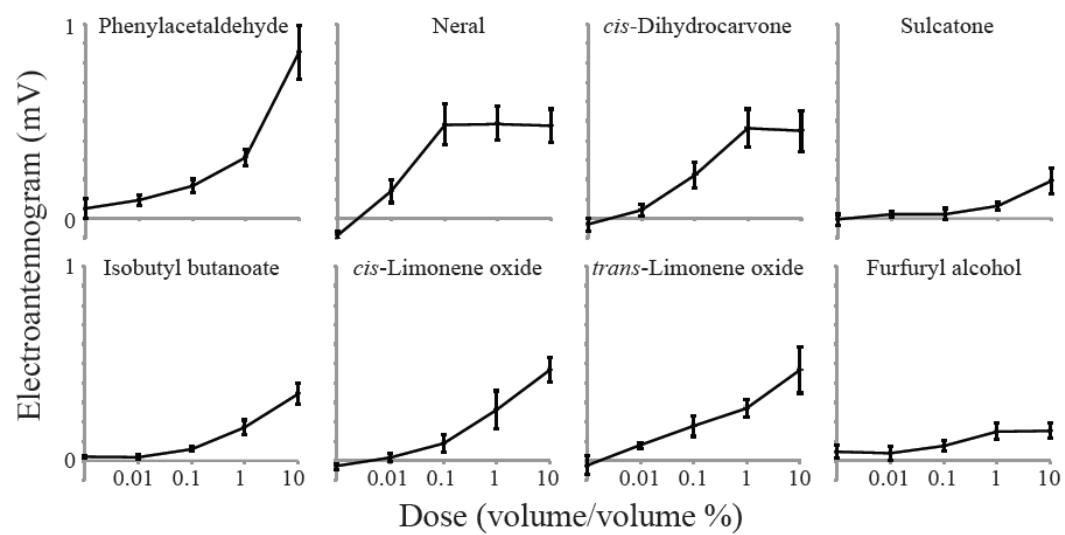

Supplement: Supplementary file 2 — 10.1186/s12936-016-1386-3 Antennal responses of female Anopheles arabiensis to various doses of compounds identified in the headspace of chicken feathers, cattle hair, goat hair and sheep wool. Error bars represent the standard error of the mean (n = 6). [file 12936_2016_1386_MOESM2_ESM.pdf]
